# Convolutional Block Attention Module–Multimodal Feature-Fusion Action Recognition: Enabling Miner Unsafe Action Recognition

**DOI:** 10.3390/s24144557

**Published:** 2024-07-14

**Authors:** Yu Wang, Xiaoqing Chen, Jiaoqun Li, Zengxiang Lu

**Affiliations:** School of Mining Engineering, University of Science and Technology Liaoning, Anshan 114051, China; wangy_sd@126.com (Y.W.); hisbur@163.com (X.C.)

**Keywords:** intelligent mine, computer vision, action recognition, multimodal feature fusion, attention mechanism

## Abstract

The unsafe action of miners is one of the main causes of mine accidents. Research on underground miner unsafe action recognition based on computer vision enables relatively accurate real-time recognition of unsafe action among underground miners. A dataset called unsafe actions of underground miners (UAUM) was constructed and included ten categories of such actions. Underground images were enhanced using spatial- and frequency-domain enhancement algorithms. A combination of the YOLOX object detection algorithm and the Lite-HRNet human key-point detection algorithm was utilized to obtain skeleton modal data. The CBAM-PoseC3D model, a skeleton modal action-recognition model incorporating the CBAM attention module, was proposed and combined with the RGB modal feature-extraction model CBAM-SlowOnly. Ultimately, this formed the Convolutional Block Attention Module–Multimodal Feature-Fusion Action Recognition (CBAM-MFFAR) model for recognizing unsafe actions of underground miners. The improved CBAM-MFFAR model achieved a recognition accuracy of 95.8% on the NTU60 RGB+D public dataset under the X-Sub benchmark. Compared to the CBAM-PoseC3D, PoseC3D, 2S-AGCN, and ST-GCN models, the recognition accuracy was improved by 2%, 2.7%, 7.3%, and 14.3%, respectively. On the UAUM dataset, the CBAM-MFFAR model achieved a recognition accuracy of 94.6%, with improvements of 2.6%, 4%, 12%, and 17.3% compared to the CBAM-PoseC3D, PoseC3D, 2S-AGCN, and ST-GCN models, respectively. In field validation at mining sites, the CBAM-MFFAR model accurately recognized similar and multiple unsafe actions among underground miners.

## 1. Introduction

More than 76% of extraordinarily severe mine accidents are attributed to human error factors, including weak safety awareness, inadequate professional competence, and ineffective safety management [1]. Miners’ unsafe behavior (MUB), one of the most prominent human error phenomena, has caused great concern. Monitoring and recognizing underground miners’ unsafe actions is carried out manually [2]. However, due to the fatigue and inadvertent negligence of monitoring personnel, the MUB is likely not monitored in time, which leads to how to ensure the safety of underground miners, which has become an urgent problem to be solved in mining production.

Using reliable technology to strengthen the recognition and control of MUB can effectively reduce the occurrence of accidents in underground mines and ensure the safe production of mining enterprises. With the rapid development of modern industry, many computer technologies, such as big data [3], cloud computing [4], the Internet of things [5], artificial intelligence [6], etc., are applied in the production and supervision of mines [7]. The emergence of computer vision technology provides a new tool for identifying the unsafe behavior of humans and preventing man-made accidents. Many research achievements on human action recognition have been carried out based on this technology [8,9], and many algorithms for human action recognition have been developed and applied to recognize MUB in underground mining. For example, Qian et al. [10] utilized an improved residual network model to detect abnormal human action. Guan et al. [11] proposed a method for recognizing unsafe actions, combining a 3D convolutional neural network and a long short-term memory network, demonstrating strong performance on specific abnormal action datasets. Chen et al. [12] integrated an adaptive self-attention mechanism and weighted feature pyramid network into a YOLO network to recognize unsafe actions such as smoking in industrial settings. Yang et al. [13] introduced the spatial–temporal relation transformer method for identifying unsafe actions on construction sites. Li et al. [14] applied an improved Faster R-CNN-LSTM network to identify unsafe actions, particularly excelling in distinguishing fuzzy actions well. Based on computer vision technology, Wen et al. [15] presented a recognition method by utilizing a residual network and transfer learning to recognize MUBs. Shi et al. [16] proposed a MUB recognition algorithm based on an improved Alphapose-STGCN, achieving the recognition accuracy of 82.3% on the coal mine underground action dataset. Wang et al. [17] applied an optimized YOLOv8 to identify non-standard miner’s actions while balancing computational complexity with recognition accuracy. Wang et al. [18] constructed a coal miner’s action-recognition model that combined spatiotemporal dual-branch structure with transposed attention representation mechanism, achieving an accuracy of 92.37% on their self-built miner action dataset.

Previous studies have made significant progress in recognizing unsafe actions by underground miners. However, action-recognition algorithms based on computer vision encounter challenges requiring resolution in underground production environments. Firstly, the poor lighting conditions, excessive dust, and susceptibility to electromagnetic interference in underground mines result in captured images with varying brightness, low contrast, and high noise levels [19], adversely affecting the accuracy of action recognition. Secondly, background clutter, miners’ clothing and gear, and occlusion between miners pose additional challenges for action recognition. Finally, relying solely on a single modality for action recognition limits feature-extraction capabilities. For example, using only skeleton modality captures human motion information but overlooks appearance details of people and objects, leading to potential misrecognition of actions. Therefore, this paper proposes a computer-vision-based method, which is carried out by the Convolutional Block Attention Module–Multimodal Feature-Fusion Action Recognition (CBAM-MFFAR) model, for identifying unsafe actions of underground miners (UAUM).

## 2. Miner Action-Recognition Methods

### 2.1. General Architecture of Miner Action-Recognition Model

**Figure 1:**
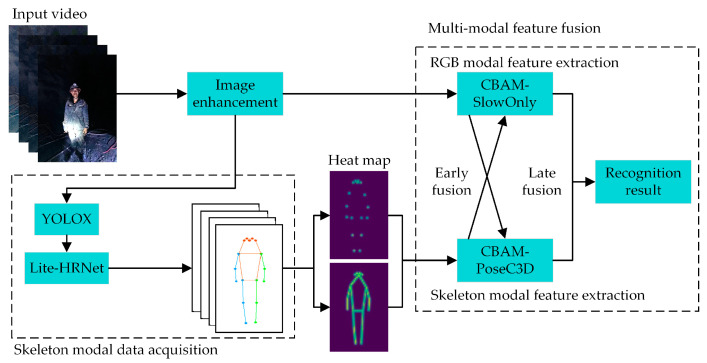
The architecture of the miner’s unsafe-action-recognition model.

In addressing the need for recognizing UAUM, the general architecture of an underground miners’ unsafe-action-recognition model is presented (Figure 1). The model consists of several components: image enhancement, acquisition of skeleton modal data, RGB modal feature extraction, skeleton modal feature extraction, and multimodal feature fusion. An image enhancement algorithm enhances video clarity from input RGB video data. At the same time, the CBAM-SlowOnly network extracts RGB modal features utilizing an improved version with a hybrid attention module [20]. To obtain the skeleton modal data of the miners, the YOLOX algorithm [21] first performs human target detection on the input RGB video data, thereby acquiring the location information of the miners in the image. Subsequently, the Lightweight High-Resolution Network (Lite-HRNet) [22] is employed to gain 2D skeleton key points yielding skeleton modal data of the miners. The obtained 2D human skeleton key points are used to generate corresponding 2D key-point heatmaps. These heatmaps are then stacked frame by frame to create a compact 3D heatmap stack and input into the CBAM-PoseC3D network with an enhanced attention module [23], extracting skeleton modal features. During this process, the CBAM-SlowOnly and CBAM-PoseC3D networks perform early fusion through bidirectional lateral connections followed by late fusion of RGB modal and skeleton modal features, resulting in the identification of the UAUMs.

### 2.2. Image Enhancement Processing

The image enhancement algorithm is applied to the images collected in an underground mine to eliminate noise, improve image clarity, and enhance edge details. Image enhancement algorithms can be classified into two major categories based on the domain in which they are performed: spatial-domain-based enhancement algorithms and frequency-domain-based enhancement algorithms. Spatial-domain enhancement algorithms include gamma correction and histogram equalization, while frequency-domain methods include Gaussian low-pass filtering.

Gamma correction [24], also known as power-law transformation, changes the image’s brightness and contrast of the pictures by altering the grayscale values of the pixels by the nonlinear transformation method. The grayscale value *g*(*x*, *y*) of the pixel after correction can be calculated by

(1) $$g\left(x,y\right)=p{\left[\frac{f\left(x,y\right)}{p}\right]}^{\gamma }$$

where *f*(*x*, *y*) is the grayscale value of any pixel point (*x*, *y*) in the original image, *γ* is the gamma value which controls the degree of the transformation, and *p* is the maximum pixel value of the image, which is 255 in this paper.

The histogram equalization method [25] is used to improve the image’s contrast. The image’s histogram with the new gray value obtained by the transformation algorithm is redistributed uniformly. The transform algorithm of the histogram equalization uses the cumulative distribution function (CDF), which can be presented as

(2) $$S_{k}=\left(L-1\right)\sum_{i=0}^{k}\frac{n_{i}}{n},k=0,1,2,\cdots ,L-1$$

where *k* is the gray level of the current pixel with a range of [0, *L* − 1], and *L* is the value of the grayscale level owned by the image and has 256 gray levels for a typical 8-bit image. So, the range of *k* is [0, 255], and that is why *p* in Formula (1) is 255. *n_i_* is the number of pixels contained in the *i*th gray level in the image, *n* is the total number of pixels in the image, and *S_k_* is the grayscale value of the transformed image pixel.

Image enhancement in the frequency domain converts the image from the spatial domain to the frequency domain by using the Fourier transform, which is different from image enhancement in the spatial domain, which directly transforms image pixel values. In the frequency domain, the image comprises components with different frequencies. When the images need to be enhanced, the image signal can be filtered to suppress part of the frequency components and achieve the enhancement purpose. Then, the image is converted back to the spatial domain by Fourier inversion, and the enhanced image is obtained. The formulas for frequency-domain enhancement are as follows

(3) q(x,y)=h(x,y)∗p(x,y)

(4) Q(u,v)=H(u,v)P(u,v)

where *h*(*x*, *y*) is the filter function; ∗ signifies the convolution operation; and *P*(*u*, *v*), *H*(*u*, *v*), and *Q*(*u*, *v*) are obtained through Fourier transformation from *p*(*x*, *y*), *h*(*x*, *y*), and *q*(*x*, *y*), respectively. After frequency-domain filtering, the image can be obtained by Fourier inversion of *Q*(*u*, *v*).

The noise in the image usually corresponds to the high-frequency components in the frequency domain, and it can be filtered by low-pass filtering to remove noise. Gaussian low-pass filtering [26] can remove Gaussian noise better in the image and retain more low-frequency information. Its formula is as follows

(5) $$D\left(u,v\right)=\sqrt{{\left(u-\frac{M}{2}\right)}^{2}+{\left(v-\frac{N}{2}\right)}^{2}}$$

(6) $$H\left(u,v\right)=e^{-\frac{D^{2}\left(u,v\right)}{2D_{0}^{2}}}$$

where *D*(*u*, *v*) is the Euclidean distance from the point (*u*, *v*) in the spectrum to the center point of the spectrum, *M* and *N* are the spectrum’s height and width, respectively, and *D*_0_ is the cutoff frequency. The larger the value of *D*_0_, the more high-frequency information can be retained after Gaussian low-pass filtering. *H*(*u*, *v*) is the Gaussian low-pass filter function.

Above all, to balance image brightness, improve image contrast, and filter noise information, the three methods, which are gamma correction, histogram equalization, and Gaussian low-pass filtering, will be combined to form a method to enhance the underground images.

### 2.3. Skeleton Modal Data Acquisition

The skeleton modal data contain the skeleton key points of the human body and can extract the relevant human motion information. Using skeleton modal data for action-recognition networks can yield better recognition results. However, due to the complex production environment and background noise interference, extracting human action data from images is difficult. The noise contained in human action data has an adverse effect on subsequent action recognition. So, to obtain 2D skeleton key-point data and generate skeleton modal data for input into the action-recognition network, the YOLOX object detection algorithm is used to detect the positions of human targets in the input RGB images. The Lite-HRNet is used to detect the human body’s key points in the object detection results.

**Figure 2:**
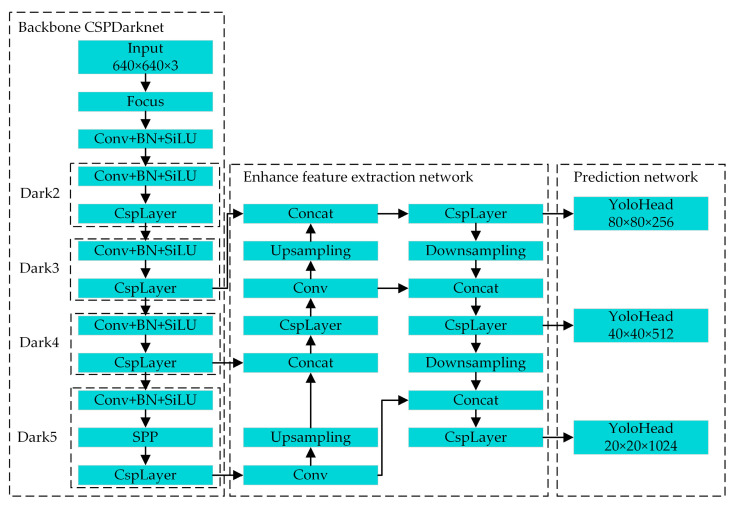
The overall architecture of the YOLOX object detection network.

The overall architecture of the YOLOX object detection network is shown in Figure 2. The network consists of the backbone, enhanced feature extraction, and prediction networks, i.e., CSPDarknet, PAFPN, and YoloHead. The backbone network is responsible for extracting the basic features of the input image. There are three layers, i.e., the Dark3, Dark4, and Dark5 layers, in the backbone network for generating three adequate feature layers at different scales, which are then input into the enhanced feature-extraction network. The improved feature-extraction network fuses the adequate feature layers of different scales. The prediction network performs the final classification and regression to obtain detection results of objects on various scales from the three enhanced adequate feature layers.

**Figure 3:**
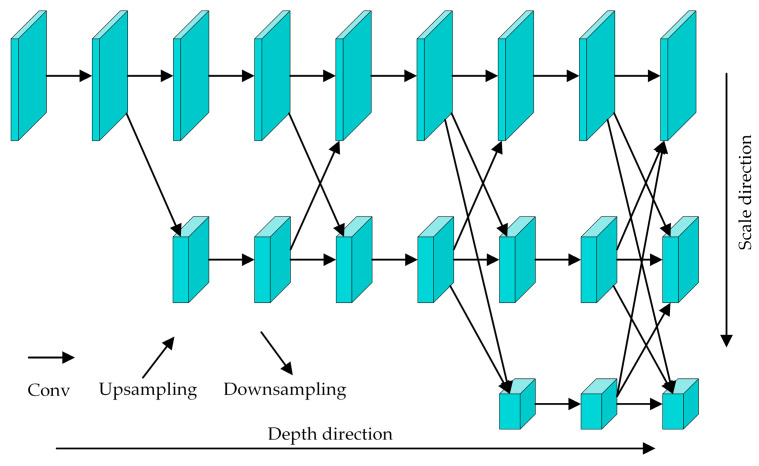
The architecture of HRNet.

High-Resolution Network (HRNet) [27] can retain the spatial information in the image better and is suitable for detecting the spatial position information, e.g., human key points. Its basic architecture is illustrated in Figure 3. HRNet maintains a high-resolution feature map branch throughout the process, including the spatial information in the original image, and obtains the other two branches of the feature maps with lower resolution by multiple downsampling. The branches of low-resolution feature maps contain more substantial semantic information via continuous convolution, and the feature fusion between different scales is carried out by upsampling. So, the deep-level semantic information of the network is transmitted to high-resolution feature maps, which contain more substantial spatial position information. The Lite-HRNet, combined Shuffle Block in the lightweight network Shufflenet [28] with the original HRNet, is a lightweight model of the HRNet and is the top-down type of pose estimation algorithm. It replaces the 1 × 1 convolution operation in the Shuffle Block with the conditional channel weighting operation. These modifications significantly reduce the computational load of Lite-HRNet, speeding up network detection. By retaining the high-resolution feature layers of HRNet, strong semantic and spatial information can be obtained, and the precision of human skeleton key-point detection can be enhanced.

### 2.4. Action-Recognition Model

Generally, the graph convolutional network (GCN) method is typically used to extract and classify the skeleton modal data from the images. Still, the skeleton modal data extracted from underground mine images lack robustness due to a certain amount of noise. In addition, the recognition speed of multiple scenes is low. Therefore, the CBAM-PoseC3D and CBAM-MFFAR models can be obtained by adding an attention enhancement module, improving the recognition accuracy of the UAUM.

#### 2.4.1. Hybrid Attention Module

**Figure 4:**
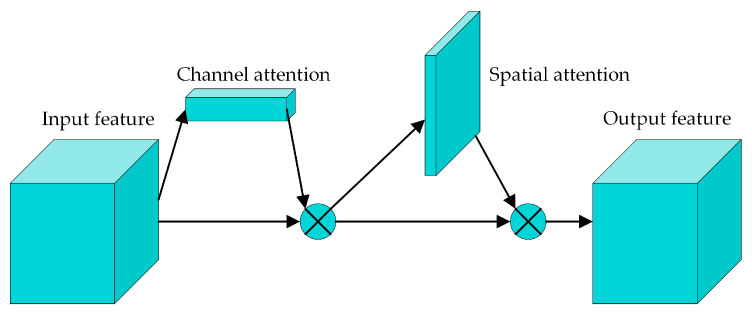
The architecture of the CBAM.

In the feature-extraction process, the attention mechanism can focus on helpful information to enhance the feature-extraction ability. The attention mechanism can be divided into channel attention, spatial attention, temporal attention, branch attention, and so on. The convolutional block attention module (CBAM) [29] is a kind of hybrid attention mechanism that combines the channel attention module (CAM) and spatial attention module (SAM), and its structure is shown in Figure 4.

The CBAM comprises two sub-modules, i.e., the CAM and the SAM, from which a channel attention feature map and a spatial attention feature map can be obtained. Then, the two attention features are multiplied element-wise with the input feature, and the output feature maps with attention enhancement can be obtained.

**Figure 5:**
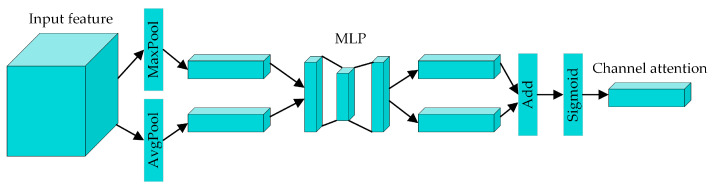
The architecture of the channel attention module.

The architecture of the CAM is shown in Figure 5. Initially, the spatial dimensions of the input features are compressed by global max pooling and average pooling, aggregating the spatial information into two different feature maps: the input into the multilayer perceptron (MLP). These two feature maps are then summed together. Finally, the output channel attention feature map can be obtained through a Sigmoid function. The corresponding formula for the CAM is

(7) $$M_{c}\left(F\right)=\sigma \left(MLP\left(MaxPool\left(F\right)\right)+MLP\left(AvgPool\left(F\right)\right)\right)$$

where *σ* represents the Sigmoid function, *MaxPool* and *AvgPool* represent the global max pooling and global average pooling operations, respectively, *F* represents the input features, and *M_c_* represents the output channel attention feature map.

**Figure 6:**
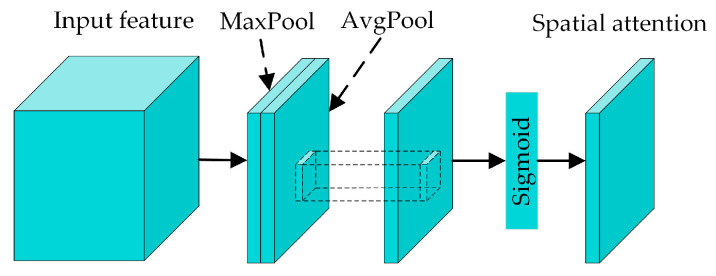
The architecture of spatial attention module.

The architecture of the SAM is shown in Figure 6. Initially, the channel dimensions of the input features are compressed into two single-channel feature maps by max and average pooling operations. These two feature maps are then concatenated, followed by a convolution operation to reduce the dimensionality, aggregating the channel information to a single-channel feature map. Finally, the output spatial attention feature map can be obtained via a Sigmoid function. The corresponding formula for the SAM is

(8) $$M_{s}\left(F\right)=\sigma \left(f^{7\times 7}\left(\left[MaxPool\left(F\right);AvgPool\left(F\right)\right]\right)\right)$$

where *f ^7×7^* denotes a convolution operation with a kernel size of 7 × 7, and *M_s_* denotes the output spatial attention feature map.

In summary, the CBAM can be easily integrated with the convolutional modules of existing feature-extraction networks to improve the feature-extraction ability of the network in channels and spatial dimensions, strengthen the overall generalization of the feature-extraction network, and improve the network’s performance.

#### 2.4.2. Construction of the CBAM-PoseC3D

PoseC3D is a model that uses a 3D convolutional neural network (3D-CNN) to process skeleton modal data for action recognition. PoseC3D, different from GCN-based methods that separately extract spatial features and temporal features from skeleton graph data, can simultaneously extract the spatial and temporal features and has spatiotemporal modeling capabilities. The other difference between the GCN and PoseC3D is the different inputs. GCN uses skeleton graph sequence data, but PoseC3D uses heatmap stack data generated from skeleton key-point data. The skeleton key-point detection algorithm is used to create the human skeleton key points from images, yielding both the key-points’ coordinates and confidence scores. The corresponding key-point heatmap is generated in Gaussian distribution with the coordinates of each key point as the center and the confidence as the maximum value. The generation formula is

(9) $$heatmap\left(x,y\right)=e^{-\frac{{\left(x-x_{k}\right)}^{2}+{\left(y-y_{k}\right)}^{2}}{2*\sigma^{2}}}*c_{k}$$

where *σ* is the standard deviation of the Gaussian distribution, (*x_k_*, *y_k_*) are the coordinates of the skeleton key point, and *c_k_* is the confidence score of the key point.

Similarly, a heatmap corresponding to the bone formed by connecting the two skeleton key points can also be generated via Gaussian distribution. The generating formula can be expressed as

(10) $$heatmap\left(x,y\right)=e^{-\frac{D{\left(\left(x,y\right),seg\left[a_{k},b_{k}\right]\right)}^{2}}{2*\sigma^{2}}}*\min\left(c_{a_{k}},c_{b_{k}}\right)$$

where *a_k_* and *b_k_* are the skeleton key points at the two ends of the bone, *D* is the distance from the point (*x*, *y*) to the line segment *seg*[*a_k_*, *b_k_*], and *c_ak_* and *c_bk_* are the confidence scores of the key points *a_k_* and *b_k_*, respectively.

**Figure 7:**
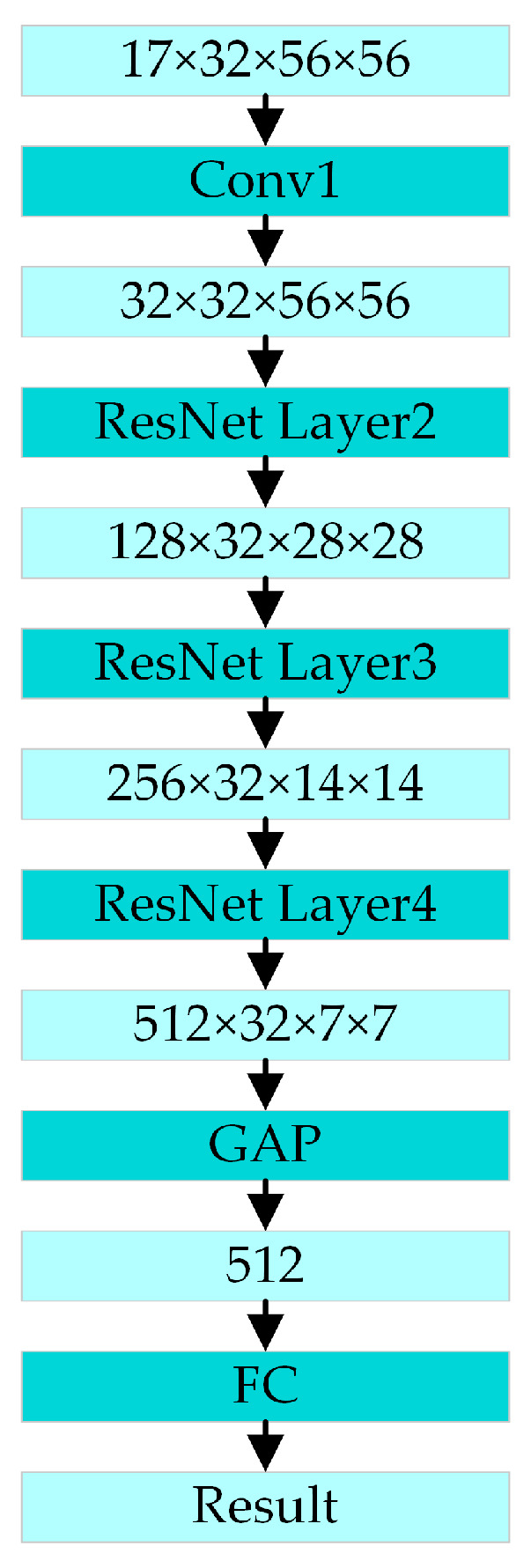
The architecture of the PoseC3D action-recognition model.

The architecture of the PoseC3D is shown in Figure 7. The input for action recognition is a stack of 3D heatmaps created by stacking 2D heatmaps generated from key-point coordinates in the temporal order of the original image frames. The shape of the 3D heatmap stack is *K* × *T* × *H* × *W*, and *T* is the number of 2D heatmaps in the stack corresponding to the number of frames in the original video. *K* is the number of skeleton key points. *H* and *W* are the height and width of the heatmaps, respectively. After multiple convolutions and multiple ResNet layers, the corresponding spatial and temporal features are extracted from the stacking of 3D heat maps. Finally, the action classification result is output through the global average pooling and a fully connected layer.

**Figure 8:**
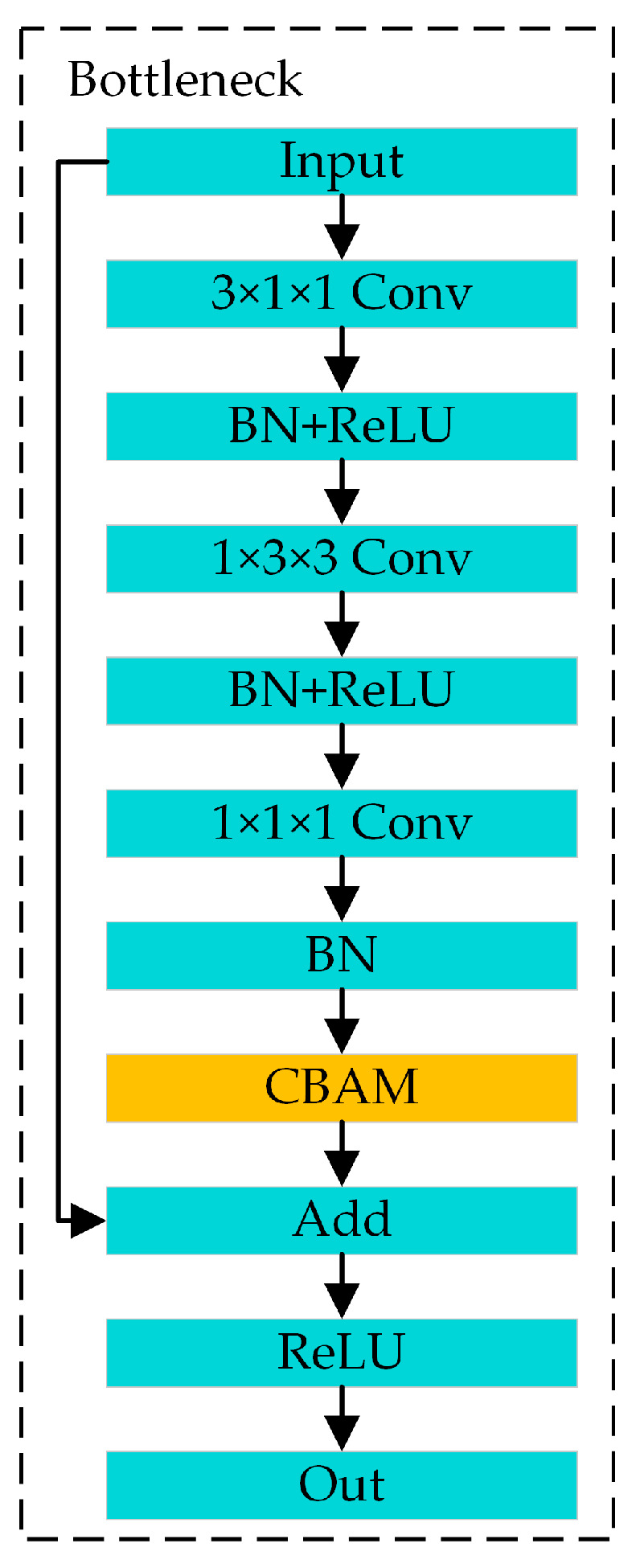
Bottleneck joining the CBAM attention module.

The CBAM-PoseC3D action-recognition model is created by embedding the CBAM module into the PoseC3D network. Specifically, the CBAM module is inserted into each bottleneck block of the PoseC3D model. The bottleneck structure after the CBAM is embedded is shown in Figure 8. The CBAM module is placed at the point where features pass through three 3D convolutions just before they are added to the residual connection. With the increase in feature-extraction network layers, feature channels also increase. Therefore, introducing an attention mechanism into the action-recognition model can enhance the model’s ability to extract the key information in spatial and channel features but only increase a small number of parameters and computation.

#### 2.4.3. Construction of the CBAM-MFFAR

**Figure 9:**
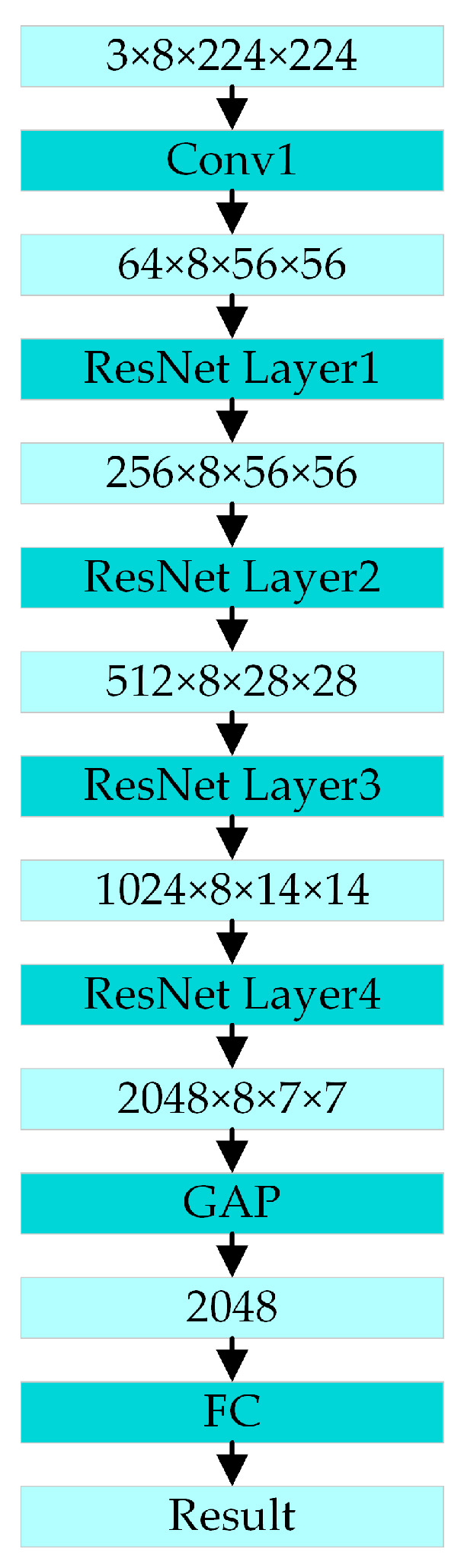
The architecture of SlowOnly.

Because the SlowOnly branch of the SlowFast network has a low video sampling frequency and more feature channels, it can effectively extract the spatial features from videos and capture sufficient object color and appearance information from the RGB modality. Its architecture is shown in Figure 9.

To further strengthen the ability to extract spatial and channel features, the CBAM module can be added to the SlowOnly network, and the addition method is similar to the CBAM-PoseC3D network used for extracting skeleton modal features, i.e., the CBAM module is embedded in each bottleneck that constitutes the SlowOnly network.

**Figure 10:**
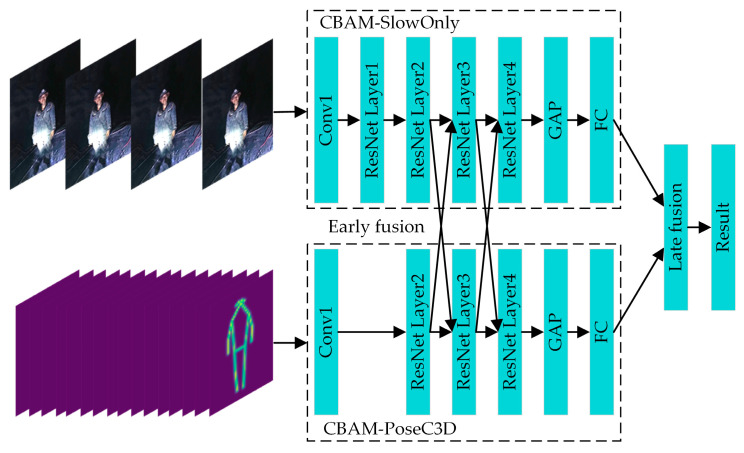
The architecture of the CBAM-MFFAR model.

Considering the characteristics of different feature-fusion methods and the practical requirements of identifying the UAUMs, the combination of early and late fusion is used to carry out the feature fusion from the RGB and skeleton modalities to form the CBAM-MFFAR model. The architecture of the CBAM-MFFAR model is shown in Figure 10.

It can be seen from Figure 10 that the CBAM-MFFAR model is composed of two branch networks, namely the CBAM-SlowOnly network, which extracts the RGB modal features, and the CBAM-PoseC3D network, which extracts skeleton modal features. The RGB modal feature-extraction branch can provide rich spatial information, including the exterior and color of objects and people; the skeleton modal feature-extraction branch can provide detailed motion information by inputting a stack of skeleton heatmaps that have a higher sampling frequency. Before training the feature-fusion model, the two branch networks were pre-trained separately. The weights obtained from the pre-training were used to initialize the CBAM-MFFAR model to improve the convergence speed of the fusion model.

The multimodal feature fusion adopts the hybrid approach of early and late fusion. The early fusion is arranged after the ResNet Layer2 and the ResNet Layer3 in the early feature-extraction stage of the model. The feature fusion between the two modalities is carried out through the bidirectional lateral connections using concatenation operations. Compared to the unidirectional lateral connections, the bidirectional lateral connections can help the entire fusion model better learn the spatiotemporal features of different modalities and make the two networks complement each other with information. The late fusion is placed at the final stage of the model; it fuses the prediction scores of the two networks and outputs the action classification results.

## 3. Action-Recognition Experiments

### 3.1. Experimental Datasets

The NTU60 RGB+D dataset [30] is a public action-recognition dataset published by Nanyang Technological University. It features 40 actors filmed and contains 60 categories and 56,880 action sample videos. There are two standards in the datasets, i.e., X-Sub and X-View. According to different actors, the X-Sub (Cross-Subject) is divided into the training set and the test set, in which the videos of 20 actors are used as the training set and those of the remaining 20 actors as the test set. Based on different camera angles, the X-View (Cross-View) is assigned the training set and test set, in which the videos from two viewpoints are used as the training set and those from the rest viewpoints as the test set.

**Figure 11:**
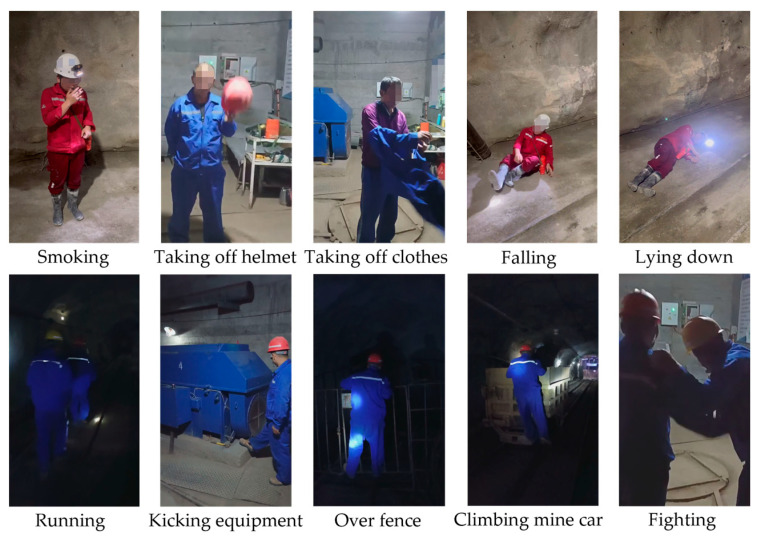
Partial sample images were selected from the UAUM dataset. Part of the images have been pixelated.

The UAUM dataset is constructed by selecting the real RGB videos from which the industrial surveillance cameras in situ are recorded at different locations in a specific underground mine. The dataset includes ten types of UAUM (as shown in Table 1). The dataset has 600 video clips in total, and each UAUM has 60 video clips. All the video clips last approximately 8 s and are recorded at a uniform frame rate of 30 fps. A total of 75% of the video clips are used as the training set, while the rest of 25% are used as the test set. Part of the image samples from the UAUM dataset are shown in Figure 11; due to privacy concerns, parts of the images have been pixelated.

### 3.2. Action-Recognition Experimental Platform and Parameters

The experimental platform, using Python 3.8 as the programming language and Pytorch version 1.10.0 as the deep learning framework, is carried out via the Ubuntu 18.04 operating system. The computing platform uses CUDA version 11.3, with an Intel Xeon Gold 6271 processor, an Nvidia Tesla P100-16G GPU, and 48 GB of RAM.

During the experiment, the two datasets, i.e., the NTU60 RGB+D dataset and the UAUM dataset, were used to test the application effect of the established UAUM recognition model. Additionally, before the experiment began, skeleton modal data of human bodies were extracted from the NTU60 RGB+D and UAUM datasets using the YOLOX and Lite-HRNet algorithms. These data were saved as pickle files to serve as inputs for the action-recognition models utilizing skeleton modal data. Four recognition models, i.e., the CBAM-PoseC3D model, the PoseC3D model, the ST-GCN model [31], and the 2S-AGCN model [32], are used in comparison with the CBAM-MFFAR model.

In the process of the experiment, the experimental parameters of the two datasets are shown in Table 2.

The experimental parameters for the NTU60 RGB+D public dataset are set as follows: The algorithm optimizer used is SGD with an initial learning rate of 0.2, adjusted using the cosine annealing algorithm. The weight decay is set to 0.0003, the momentum value is 0.9, the batch size is 8, and the training is conducted for 240 epochs.

The experimental parameters for the UAUM dataset are as follows: The algorithm optimizer used is SGD, with an initial learning rate of 0.1. The learning rate is adjusted using the cosine annealing algorithm, with weight decay set to 0.0001 and momentum value set to 0.9. The batch size is 8, and the training runs for 160 epochs.

### 3.3. Experimental Results

To evaluate the reliability of the established UAUM recognition model, the accuracy evaluation index *A* was used in the action-recognition experiments. It can be calculated by

(11) $$A=\frac{n}{N}$$

where *n* is the number of samples with correct prediction, and *N* is the total number of samples.

#### 3.3.1. Experimental Results Based on the NTU60 RGB+D Public Dataset

**Figure 12:**
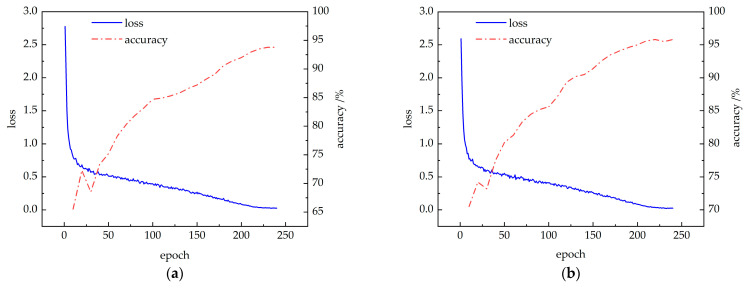
Model training accuracy and loss curves: (**a**) CBAM-PoseC3D; (**b**) CBAM-MFFAR.

The improved CBAM-PoseC3D and CBAM-MFFAR models were trained on the publicly available NTU60 RGB+D dataset under the X-Sub standard, using the specified experimental parameters. The changes in training loss and validation accuracy are shown in Figure 12. It can be observed that with the increase in training epochs, the loss values for both models decrease and eventually stabilize. Simultaneously, the accuracy of the CBAM-PoseC3D and CBAM-MFFAR models gradually increases, converging at 93.8% and 95.8% at the 230th and 220th epochs, respectively.

The established UAUM recognition model, i.e., the CBAM-MFFAR model, and the other four recognition models, were used to recognize the action based on the NTU60 RGB+D dataset under the X-Sub standard. The accuracy of the action-recognition results was calculated by Formula (11); the results are shown in Table 3.

It can be seen from Table 3 that the established CBAM-MFFAR model achieved recognition accuracy of 95.8%; compared with the CBAM-PoseC3D, PoseC3D, 2S-AGCN, and ST-GCN models, the recognition accuracy increased by 2%, 2.7%, 7.3%, and 14.3%, respectively. The results show that the action-recognition model, which compromises the modal features of both RGB and the human skeleton, achieved a higher recognition accuracy than that of the single skeleton modal feature, proving that the fusion of features among the multiple modalities effectively enhances the interaction of spatiotemporal information, and validated the effectiveness and practicality of the fusion approach.

To validate the effect of the different feature-fusion methods and the addition of the CBAM attention module on the recognition accuracy of the CBAM-MFFAR model, a comparative experiment was carried out with various combinations of early fusion, late fusion, and the attention module. The experimental results are shown in Table 4.

It can be seen from Table 4 that the combination of the CBAM attention module with early fusion and late fusion achieved the highest recognition accuracy of 95.8%. This represents an improvement of 0.4% over the early fusion + late fusion without the attention module, 0.6% over the CBAM + late fusion without early fusion, and 1% over the model using only late fusion. These results indicate that the feature fusion method combining early and late fusion is superior to using only late fusion. Additionally, the addition of the attention module to the branch networks effectively enhances the recognition accuracy of the CBAM-MFFAR model.

#### 3.3.2. Experimental Results Based on the UAUM Dataset

**Figure 13:**
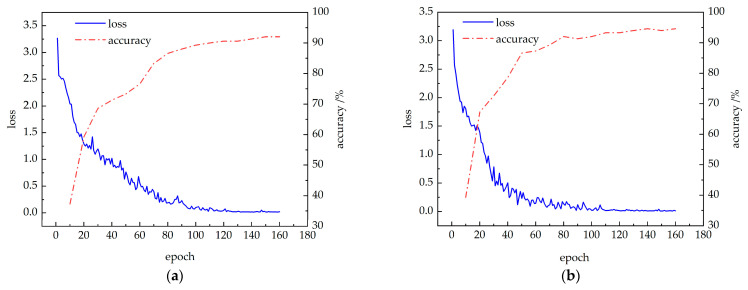
Model training accuracy and loss curves: (**a**) CBAM-PoseC3D; (**b**) CBAM-MFFAR.

The CBAM-PoseC3D and CBAM-MFFAR models were validated on the UAUM dataset, with the training results shown in Figure 13. It can be seen that the loss values of each recognition model rapidly decrease at the beginning of the training and gradually stabilize. Simultaneously, the recognition accuracy of the CBAM-PoseC3D and CBAM-MFFAR models also increases rapidly after the start of training, converging at 92.0% and 94.6% at the 150th and 140th epochs, respectively.

Similarly, the five recognition models used in the experiments described in 3.2.1 were also used to recognize the actions based on the established dataset of UAUM, and the accuracies of the recognition results are shown in Table 5.

It can be seen from Table 4 that the CBAM-MFFAR model achieved the highest recognition accuracy of 94.6%. Compared with that of the CBAM-PoseC3D, PoseC3D, 2S-AGCN, and ST-GCN models, it increased by 2.6%, 4.0%, 12.0%, and 17.3%, respectively. Compared to the results shown in Table 4, which are carried out based on the NTU60 RGB+D dataset, the recognition accuracy of each recognition model has decreased because the UAUM is more complex and difficult to identify than the routine action in the public dataset. The results indicate that the action-recognition models based on 3D-CNN have higher recognition accuracy than those based on GCN. The hybrid attention mechanism can effectively extract useful features from the UAUMs and improve recognition accuracy. Moreover, compared to the action-recognition models based on a single skeleton modality, the CBAM-MFFAR model can obtain a higher recognition accuracy and be suitable for recognizing the UAUMs.

**Figure 14:**
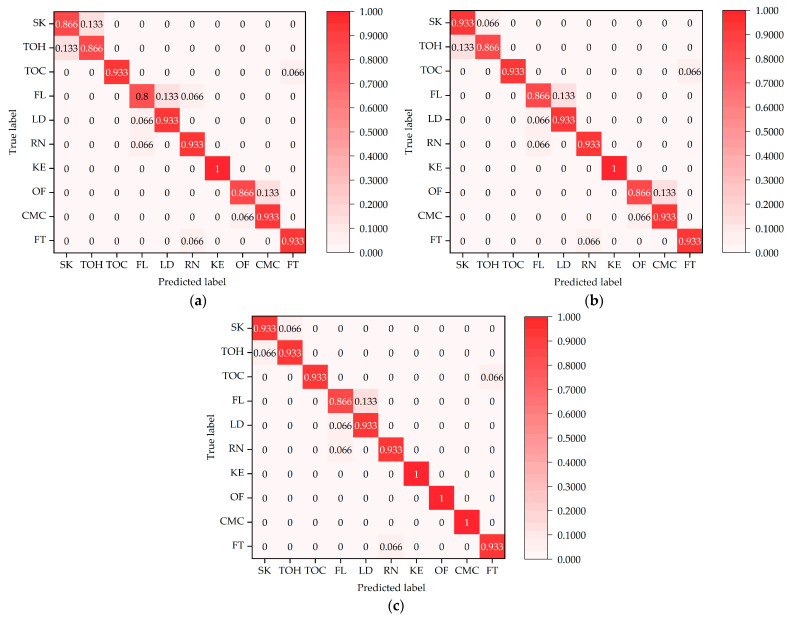
Normalized confusion matrices of the recognition models: (**a**) normalized confusion matrix of PoseC3D; (**b**) normalized confusion matrix of CBAM-PoseC3D; (**c**) normalized confusion matrix of CBAM-MFFAR.

The normalized confusion matrices for the three action-recognition models, i.e., PoseC3D, CBAM-PoseC3D, and CBAM-MFFAR, were evaluated on the test set of the UAUM dataset, as shown in Figure 14.

It can be seen from Figure 14 that, compared to the PoseC3D and CBAM-PoseC3D models, the CBAM-MFFAR model exhibits higher accuracy in recognizing various unsafe actions. Moreover, CBAM-MFFAR is able to accurately recognize actions involving similar climbing actions, such as “over fence” and “climbing mine car”, whereas PoseC3D and CBAM-PoseC3D both exhibit misclassification issues. This indicates that compared to the single skeleton modality employed by PoseC3D and CBAM-PoseC3D, the multimodal feature fusion approach utilized by CBAM-MFFAR is more effective for recognizing unsafe actions of underground miners.

### 3.4. Recognition Results of Miner Unsafe Action

**Figure 15:**
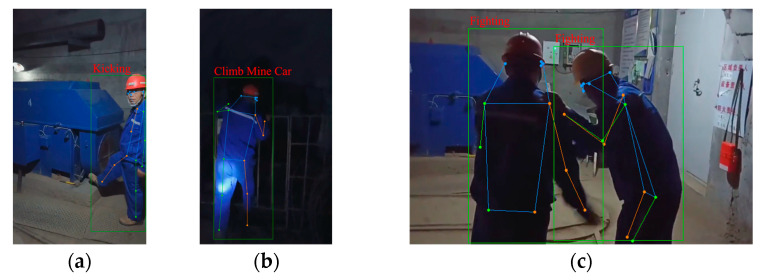
Action-recognition results: (**a**) kicking equipment; (**b**) climbing over the fence (misidentified); (**c**) fighting.

The partial recognition results of the UAUM using the improved CBAM-PoseC3D model are shown in Figure 15. It can be seen that the “kicking equipment” action in a single-person scene and the “fighting” action in a multi-person scene can be accurately identified. However, the “over fence” action was misidentified as the “climbing mine car” action since the two actions involved the climbing motions. The CBAM-PoseC3D model, based on a single skeleton modality, makes it easy to misrecognize similar actions due to the lack of corresponding exterior and color information.

**Figure 16:**
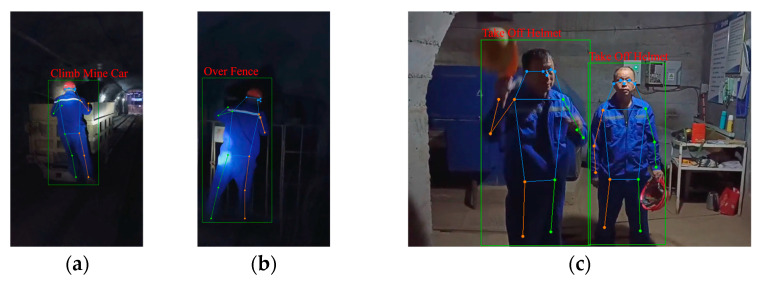
Action-recognition results: (**a**) climbing mine car; (**b**) climbing over fence; (**c**) taking off helmet.

Partial recognition results by using the CBAM-MFFAR model to recognize the UAUMs are shown in Figure 16. It can be seen that the “over fence” and the “climbing mine car” actions, which involve similar climbing motions and are misidentified by the CBAM-PoseC3D model, are accurately recognized. In multi-person identification scenarios, the action of multiple people taking off their safety helmets can also be accurately identified.

## 4. Conclusions

To realize the intelligent control of the UAUMs and reduce the accidents caused by them, a computer-vision-based recognition model of the UAUM, i.e., CBAM-MFFAR, is established in this paper. In the model, spatial- and frequency-domain enhancement algorithms enhance the images collected from underground mines. An algorithm combining the object detection algorithm (YOLOX) and the human key-point detection algorithm (Lite-HRNet) is used to obtain the human skeleton modal data. The skeleton modality action-recognition model (CBAM-PoseC3D) integrating an attention mechanism is proposed by combining the RGB modal feature-extraction model (CBAM-SlowOnly), resulting in the CBAM-MFFAR model.

To test the feasibility and reliability of the established CBAM-MFFAR model for recognizing the UAUMs, based on the public NTU60 RGB+D dataset published by Nanyang Technological University, and the UAUM dataset constructed by selecting the real RGB videos from on-site industrial photography of a mine, the established CBAM-MFFAR model is used to identify the routine actions and the UAUM; meanwhile, the four models, i.e., the CBAM-PoseC3D model, the PoseC3D model, the ST-GCN model and the 2S-AGCN model are applied in comparison. It is found that the CBAM-MFFAR model achieved the highest recognition accuracy of 95.8% and 94.6% on the X-Sub standard of the NTU60 RGB+D dataset and the UAUM dataset, respectively. It shows that the proposed model can be well applied to recognizing the UAUMs.

In future research, the following aspects are worth exploring further: Firstly, new UAUM categories can be added based on actual underground conditions, more unsafe action videos can be collected, and the scale of the UAUM dataset can be expanded. Secondly, research on the lightweight of the CBAM-MFFAR model can be conducted to improve the speed of action recognition while ensuring detection accuracy. Lastly, sensors can collect more data from various visual and non-visual modalities, such as depth, infrared, point cloud, and acceleration data. The CBAM-MFFAR model can integrate feature information from these additional modalities to further enhance the accuracy of recognizing miners’ unsafe actions.

## Figures and Tables

**Table 1:** Ten types of UAUM and their action meanings in the constructed UAUM dataset.

Action Categories	Action Meanings
Smoking (SK)	Smoking in the work area.
Taking off helmet (TOH)	Taking off the helmet in the work area.
Taking off clothes (TOC)	Taking off work clothes in the work area.
Falling (FL)	Injury from a fall.
Lying down (LD)	Sleeping in the work area.
Running (RN)	Running and chasing in the operation process.
Kicking equipment (KE)	Illegal kicking of operational equipment.
Over fence (OF)	Climbing over the fence.
Climbing mine car (CMC)	Climbing the moving mine car
Fighting (FT)	Fighting and brawling

Note: Each action category is followed by its abbreviation in parentheses for ease of subsequent use.

**Table 2:** Parameters were used in the experiment using different recognition models based on the NTU60 RGB+D dataset and the UAUM dataset.

Experimental Parameters	Dataset	
	NTU60 RGB+D	UAUM
Initial learning rate in SGD	0.2	0.1
Weight decay	0.0003	0.0001
Momentum value	0.9	0.9
Batch size	8	8
Training rounds	240	160

**Table 3:** The accuracy results of action recognition with different models based on the NTU60 RGB+D dataset under the X-Sub standard.

Recognition Model	Accuracy/%
ST-GCN	81.5
2S-AGCN	88.5
PoseC3D	93.1
CBAM-PoseC3D	93.8
CBAM-MFFAR	95.8

**Table 4:** Comparative experimental results of different combinations.

Method	Accuracy/%
Late fusion	94.8
Early fusion + Late fusion	95.4
CBAM + Late fusion	95.2
CBAM + Early fusion + Late fusion	95.8

**Table 5:** The accuracy results of action recognition with different models based on the UAUM dataset.

Recognition Model	Accuracy/%
ST-GCN	77.3
2S-AGCN	82.6
PoseC3D	90.6
CBAM-PoseC3D	92.0
CBAM-MFFAR	94.6

## Data Availability

The detailed data are available upon request.
